# Long-Term *In Vivo* Imaging of Fibrillar Tau in the Retina of P301S Transgenic Mice

**DOI:** 10.1371/journal.pone.0053547

**Published:** 2012-12-31

**Authors:** Christian Schön, Nadine A. Hoffmann, Simon M. Ochs, Steffen Burgold, Severin Filser, Sonja Steinbach, Mathias W. Seeliger, Thomas Arzberger, Michel Goedert, Hans A. Kretzschmar, Boris Schmidt, Jochen Herms

**Affiliations:** 1 Department of Translational Brain Research, DZNE – German Centre for Neurodegenerative Diseases, Munich, Germany; 2 Center for Neuropathology, Ludwig-Maximilian-University, Munich, Germany; 3 Division of Ocular Neurodegeneration, Centre of Ophthalmology, Institute for Ophthalmic Research, University of Tuebingen, Tuebingen, Germany; 4 Medical Research Council Laboratory of Molecular Biology, Cambridge, United Kingdom; 5 Clemens Schöpf-Institute of Chemistry and Biochemistry, Technische Universität Darmstadt, Darmstadt, Germany; Boston University School of Medicine, United States of America

## Abstract

Tauopathies are widespread neurodegenerative disorders characterised by the intracellular accumulation of hyperphosphorylated tau. Especially in Alzheimer's disease, pathological alterations in the retina are discussed as potential biomarkers to improve early diagnosis of the disease. Using mice expressing human mutant P301S tau, we demonstrate for the first time a straightforward optical approach for the *in vivo* detection of fibrillar tau in the retina. Longitudinal examinations of individual animals revealed the fate of single cells containing fibrillar tau and the progression of tau pathology over several months. This technique is most suitable to monitor therapeutic interventions aimed at reducing the accumulation of fibrillar tau. In order to evaluate if this approach can be translated to human diagnosis, we tried to detect fibrillar protein aggregates in the post-mortem retinas of patients that had suffered from Alzheimer's disease or Progressive Supranuclear Palsy. Even though we could detect hyperphosphorylated tau, we did not observe any fibrillar tau or Aß aggregates. In contradiction to previous studies, our observations do not support the notion that Aβ or tau in the retina are of diagnostic value in Alzheimer's disease.

## Introduction

Intracellular inclusions of hyperphosphorylated tau protein are the defining pathological hallmark of neurodegenerative disorders called tauopathies [1]. In normal brain, tau is localized in axons and plays an important role in the assembly and stabilization of microtubules [2], [3]. Under pathological conditions, tau is also found in the somatodendritic compartment in a hyperphosphorylated state, promoting the aggregation of tau to form neurofibrillary tangles (NFTs). The identification of disease-causing mutations in the microtubule associated protein tau (MAPT) has established that tau dysfunction is sufficient to cause neurodegeneration and dementia [4].

Among tauopathies, Alzheimer's disease (AD) is the most prominent form: In 2010, AD affected more than 50% of the estimated 35.6 million people worldwide suffering from dementia. Currently used diagnostic tools include cognitive tests, neuroimaging and measurement of Amyloid-beta (Aβ) or tau levels in the cerebrospinal fluid [5]. While modern imaging techniques like MRI or PET evolve to be the new hope for the diagnosis of AD, biomedical science focuses on the design of new molecular probes for the specific labelling of biomarkers like Aβ and tau *in vivo* [6], [7], [8]. However, imaging techniques like PET are restricted to specialised clinics with cost-intensive equipment and the definitive diagnosis of AD still depends on the *post mortem* analysis of the brain. This fact imposes severe restrictions on an early intervention, which is widely acknowledged to be necessary for a positive outcome of therapeutic treatments. Therefore, novel techniques and reliable biomarkers are needed for early diagnosis prior to the onset of cognitive decline [5].

The retina as part of the central nervous system is easily accessible for widely-used imaging techniques like scanning laser ophthalmoscopy (SLO) and optical coherence tomography (OCT). Therefore, pathological alterations in the retina are discussed as potential biomarkers for the diagnosis of AD. Prominent examples are the reduced retinal nerve fibre layer thickness or the decrease in retinal blood flow rate and venous diameter of AD patients [9], [10], [11], [12]. Another important biomarker could be the accumulation of Aβ-plaques within the retina like proposed by recent publications [13], [14], [15], [16].

In this study, we focused on the tau pathology in the retina. We chose the human P301S tau transgenic mouse line, a well established model of frontotemporal dementia with parkinsonism linked to chromosome 17 (FTDP-17). This model develops severe tau-pathology throughout the nervous system including the retina [17], [18], [19], [20]. Using SLO, we were able to monitor the progressing accumulation of fibrillar tau aggregates in the P301S retina over several months. We further show that hyperphosphorylated tau accumulates in the retina of patients with AD and Progressive Supranuclear Palsy (PSP). Since the examined retinas showed no fibrillar tau aggregates or Aβ-plaques, these biomarkers are of limited value for an ophthalmic diagnosis of human tauopathies.

## Materials and Methods

### Ethics Statement

The mouse studies were carried out in accordance with an animal protocol approved by the Ludwig-Maximilians-University Munich and the government of Upper Bavaria (Az. 55.2-1-54-2531-188-09). *In vivo* imaging was performed under anesthesia, and all efforts were made to minimize suffering of the animals. The use of human tissue samples was approved by the institutional review board of the Ludwig-Maximilians-University Munich (Brain-Net: Brain Banking Centre Munich – Project 068/00). Patients provided written informed consent before the tissue samples were collected and used for investigational purposes.

### Animals

We used homozygous mice expressing human mutant P301S tau [17] that were backcrossed for at least 7 generations to obtain animals on a pure C57Bl/6 background. Age-matched C57Bl/6 wild-type mice served as controls. P301S mice were further crossed with animals expressing yellow fluorescent protein (YFP) in a subset of retinal ganglion cells (RGCs) (strain B6.Cg-Tg(Thy1-YFPH)2Jrs/J, The Jackson Laboratory, Bar Harbor, USA). These mice were backcrossed to obtain animals homozygous for P301S tau x Thy1-YFPH. All groups used in this study were of mixed gender.

### Human subjects

Retina specimens from subjects with a neuropathologically confirmed diagnosis of AD (n = 6) or PSP (n = 2) and of healthy controls (n = 4) were collected (Table 1). The staging of AD specimens was performed in routine analysis of *post mortem* tissue by a experienced team of neuropathologists according to the Braak & Braak and CERAD staging [21], [22].

**Table 1:** Examined human cases.

Case	Disease	Staging (Braak& Braak, CERAD)	Age, yr/Sex	PI	AT8 Retina
1	AD	VI, C	39/M	7 h	+
2	AD	VI, C	73/F	20 h	+
3	AD	VI, C	85/M	27 h	+
4	AD	VI, C	56/F	22 h	+
5	AD	VI, C	37/M	24 h	+
6	AD	V, C	79/M	72 h	−
7	PSP	-	67/M	40 h	++
8	PSP	-	84/F	8 h	++
9	Control	0	53/M	24 h	−
10	Control	0	56/M	72 h	−
11	Control	I	60/W	18 h	−
12	Control	I	57/M	16 h	−

AD, Alzheimer's disease; F, female; M, male; PI, *post mortem* interval; PSP, Progressive supranuclear palsy; −, no AT8-positive cells; +, occasional AT8-positive cells; ++, many AT8-positive cells.

### In vivo scanning of the mouse retina

The ophthalmic examinations of the mouse retinas were performed using a modified Spectralis HRA + OCT system (Heidelberg Engineering, Dossenheim, Germany) with two different laser wavelengths for the excitation of fluorophores (450 and 488 nm) and an integrated set of emission filters (LP 458 nm, BP 550/49 nm and BP 617/73 nm). Mice were anesthetized with an intraperitoneal injection of ketamin (0.14 mg/g) and xylazin (0.01 mg/g), followed by the dilation of their pupils with Tropicamid eye drops (Mydriadicum Stulln, Pharma Stulln GmbH, Stulln, Germany). During the scanning procedure, a custom-made contact lens in combination with hydroxylpropyl methylcellulose (Methocel 2%; OmniVision, Puchheim, Germany) kept the eye moist and negated the refractive power of the interface between air and cornea [23]. A custom-made mouse holder allowed the realignment of the animal and retina for long-term examinations and the suppression of moving artefacts. Images were recorded in high resolution mode with the scanner set to 30° field of view.

### In vivo examination of FSB- and YFP-positive cells

Cells containing fibrillar tau were labelled *in vivo* by the fluorophore FSB ((*E, E*)-1-fluoro-2,5-*bis*(3-hydroxycarbonyl-4-hydroxy)styrylbenzene; Merck; product # 344101) [24]. 24–48 hours before each scanning session, mice received an i.p. injection of 10 mg/kg FSB dissolved in 10% DMSO and 90% PBS containing 2% mouse albumin (Merck; product # 126674). FSB-positive cells were excited at 450 nm (detection LP 458 nm), YFP-positive cells at 488 nm (detection BP 550/49) and spots of increased autofluorescence at 488 nm (detection BP 617/73).

### Immunohistochemistry of murine tissue

Mice were transcardially perfused with PBS and 4% PFA before the preparation of the retinal whole mounts and the post-fixation in 4% PFA for 30 min. The retinas were permeabilized in PBS containing 2% Triton-X over night and non-specific epitopes were blocked by incubating the sections with Casein I-Block for 1 h (Applied Biosystems, product # T2015). For immunohistochemical stainings, the retinal whole mounts were treated over night with the primary antibodies AT8 (1∶200; Thermo Scientific, product # MN1020), AT100 (1∶200; Thermo Scientific, product # MN1060) or anti-NeuN (1∶300; Millipore, product # MAB377). After washing 3×10 min with PBS, a secondary anti-mouse antibody conjugated with Alexa647 (1∶200; Invitrogen, product # A-21236) was applied for 4 h, followed by washing 3×10 min in PBS, the co-staining with FSB (0.001% in 50% ethanol) for 30 min and a last washing step 3×10 min in PBS.

### Immunohistochemistry of human tissue

Human eyes were obtained at autopsy and fixed in 4% formalin in PBS for 3–5 days. Afterwards, the tissue was embedded in paraffin and 2–4 µm thick sections were cut. Sections were deparaffinised and boiled for 30 min in 10 mM citrate buffer (pH 6.0) (AT8 staining) or incubated 2 min in formic acid (4G8 staining) for antigen retrieval. After blocking 30 min with Casein-I, probes were stained with antibodies against hyperphosphorylated tau (AT8, 1∶200; AT100, 1∶200; AT180, 1∶50, Thermo Scientific, product # MN1040; AT270, 1∶200, Thermo Scientific, product # MN1050 or PHF-1, 1∶1000 [25]) and Aβ (4G8, 1∶1000; Calbiochem, product # NE1002). Further, sections were treated with biotin-conjugated anti-mouse immunoglobulins and the staining was visualized by a horseradish peroxidase-diaminobenzidine reaction using the Multilink-detection kit HRP/DAB (BioGenex, product # QD-200 OX).

For the immunofluorescence staining, sections were deparaffinised and boiled like described. Sections were blocked with the endogenous biotin blocking kit (Invitrogen, product #E21390) and 30 min in Casein-I. Afterwards, the probes were treated with biotin-labelled AT8 (1∶200, Thermo Scientific, product #MN1020B) over night. After washing 3×10 min in PBS the staining was completed using the TSA-KIT with HRP-Streptavidin and Tyramid Alexa 647 (Invitrogen, product #T20936) according to the manufacturer's recommendation. After a further washing step, sections were co-staining with FSB (0.001% in 50% ethanol) for 30 min and washed 3×10 min in PBS. Alternatively, sections were co-stained with Thioflavin-S (1% in PBS) for 10 min and washed in 100% EtOH, 70% EtOH, 50% EtOH (5 min each wash) and PBS (3×10 min). Adjacent Sections were silver-impregnated using the method of Gallyas to visualize fibrillar tau pathology.

### Microscopy

Fluorescence images were acquired with a confocal laserscanning microscope (LSM 510, Carl Zeiss MicroImaging GmbH, Jena, Germany). The fluorophores were separated according to their excitation and emission properties: FSB was excited at 458 nm (detection LP 475 nm), YFP at 514 nm (detection LP 530 nm) and Alexa647 at 633 nm (detection LP 650 nm). FSB-positive cells within the retinal whole mounts were imaged with a completely opened pinhole, to ensure the detection of all cells on different levels of the retinal ganglion cell layer (GCL).

### Quantification and statistical analysis

During the long-term *in vivo* examinations, images were realigned and all observed FSB-positive cells within the scanning field of 30° were counted. Areas not present at all time points were excluded from the analysis. Curves display the mean values of all analyzed retinas at specific time points. For the *ex vivo* analysis, RGCs co-stained with AT8 and FSB were counted and normalized to the area of the retinal tissue. Each data point in the quantifications represents one retina. The statistical analysis was performed with GraphPad Prism 5.0b (GraphPad Software, San Diego, CA). To confirm statistical significance, we conducted repeated measures ANOVA test on *in vivo* data and ANOVA on *ex vivo* data. Results are shown as mean values +/− SD.

## Results

### In vivo detection of fibrillar tau aggregates in the retina of P301S mice

**Figure 1:**
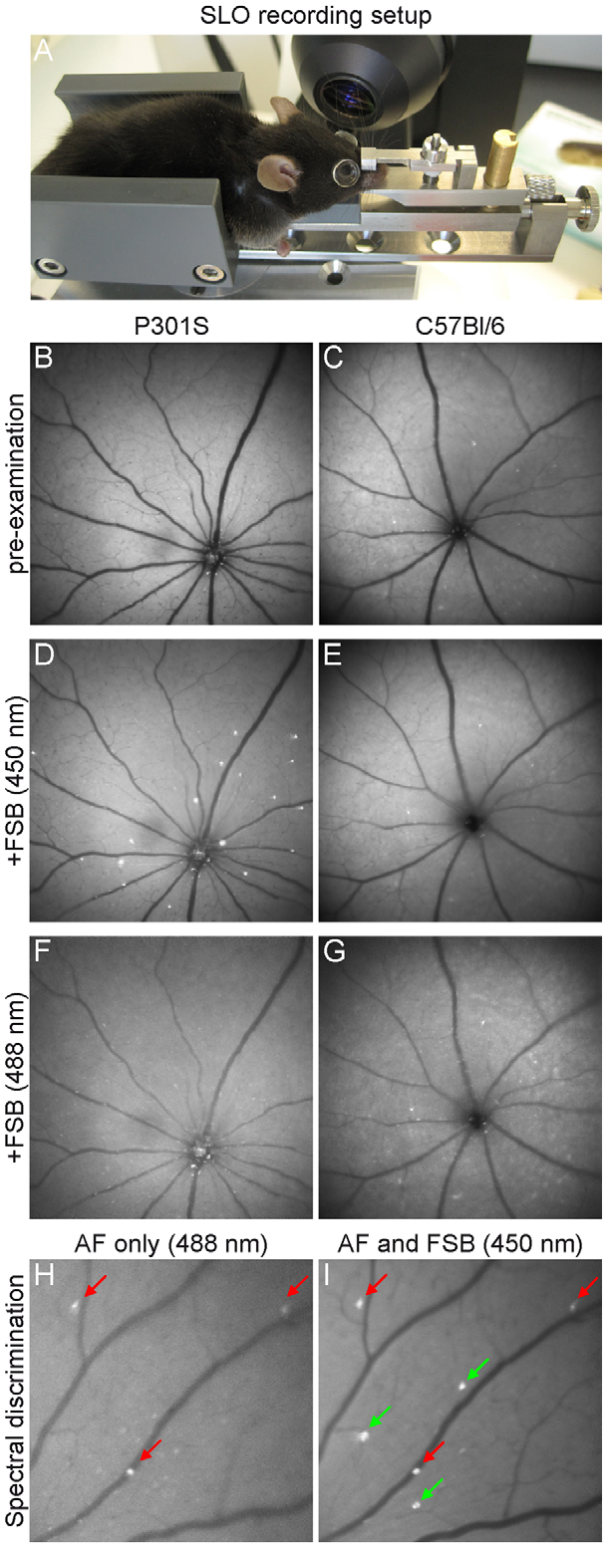
*In vivo* imaging of fibrillar tau aggregates in the retina of P301S mice. **A** SLO examination of the mouse retina using a custom made mouse holder. **B, C** Pre-examination revealing spots of increased autofluorescence in the retinas of 5-month-old P301S and C57Bl/6 mice. **D** 24–48 hours after the injection of FSB, fluorescent cells were detected in the retinas of P301S mice but not of C57Bl/6 mice at 450 nm excitation (**E**). **F, G** No FSB-positive signals could be detected in the same imaging session when excited at 488 nm. **H, I** Example for the spectral discrimination of FSB-positive cells from spots of increased autofluorescence in the retina of a P301S mouse. **H** *In vivo* image obtained at 488 nm excitation showing dots of increased autofluorescence (AF, red arrows). **I** FSB-positive cells can only be excited at 450 nm (green arrows).

Mice transgenic for the human P301S tau mutation develop fibrillar inclusions of hyperphosphorylated tau protein within cells of the GCL [18]. Using a modified SLO in combination with a custom-made mouse holder (Fig. 1A), we were able to detect these fibrillar tau aggregates in the retinas of living P301S mice. Fluorescent signals could be imaged 24–48 hours after the systemic administration of the fluorophore FSB in aged P301S animals (Fig. 1D). In comparison, no FSB-positive signal could be observed in the retina of age-matched wild-type mice (C57Bl/6, n = 3) (Fig. 1E). A major drawback for the examination of fluorescent dyes within the retina are dots of increased autofluorescence which are very prominent in the mouse retina when excited at 488 nm [26], [27]. To exclude false-positive signals emanating from autofluorescence, we examined the eyes prior to the injection of FSB (Fig. 1B, C). Furthermore, the FSB signal can be differentiated from autofluorescence by its spectral properties; FSB is limited to efficient excitation at 450 nm, whereas spots of autofluorescence became apparent upon excitation at 450 or 488 nm (Fig. 1 H, I).

**Figure 2:**
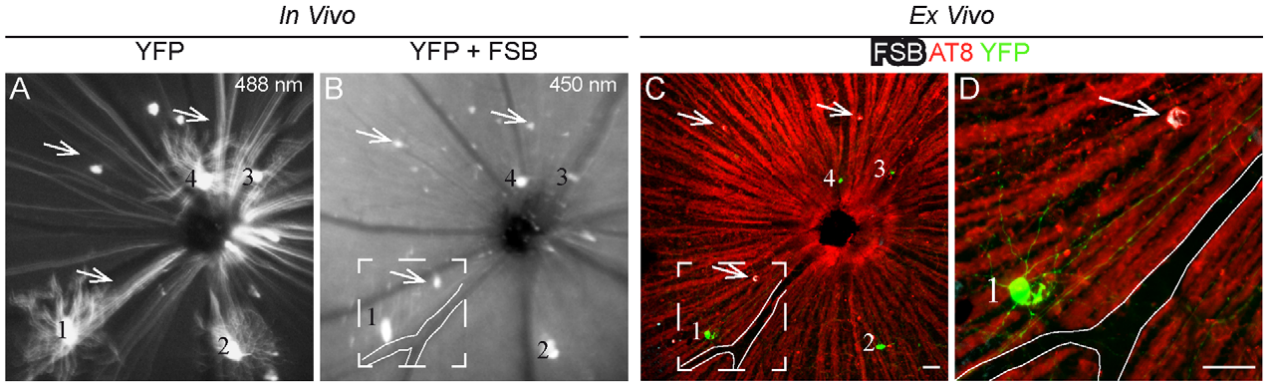
FSB labels cells containing hyperphosphorylated tau in the GCL of P301S mice. **A, B** *In vivo* SLO-examination of a P301S mouse crossed with Thy1-YFPH. **A** Scanning at 488 nm excitation revealed only YFP-positive cells in a unique pattern (Striking cells are numbered 1–4). **B** In the same retinal area, FSB-positive cells (arrows) adjacent to YFP-positive cells became apparent when excited at 450 nm. **C** Whole mount preparation of the *in vivo* examined retina (**A, B**). **D** Magnification of the boxed area in **B** and **C**. The *in vivo* observed FSB-positive cells (**B**) could be counterstained with AT8 (arrows). Axons from RGCs containing hyperphosphorylated tau appear as red background. red, AT8; white, FSB; green, YFP. Scale bars: 50 µm.

To further confirm the *in vivo* observed FSB-positive spots as cells containing fibrillar tau aggregates, we crossed P301S with Thy1-YFPH mice that express YFP in RGCs. In retinal whole mounts, FSB-positive cells were counterstained with antibodies against hyperphosphorylated tau and localized in the cell layer containing the YFP-positive RGCs (Fig. S1 A–A''''). 1.9% of the RGCs labelled with FSB were also YFP-positive. No pathological alterations could be observed in their dendritic arbors. *In vivo*, Thy1-YFPH mice show a sparse labelling of YFP-expressing ganglion cells with a unique pattern for the identification of discrete RGCs [28], [29]. After the administration of FSB, the SLO imaging revealed FSB-positive cells adjacent to YFP-positive cells (Fig. 2A, B). The two fluorophores can be separated based on their spectral properties: YFP can be excited at 450 and 488 nm, while FSB can only be detected at 450 nm. In addition, retinal whole mounts of the same mice were prepared for the relocation of the *in vivo* observed FSB-positive cells based on the unique pattern of the ganglion cells containing YFP. A consecutive counterstaining with the antibody AT8 definitely identified the *in vivo* observed FSB-positive spots (Fig. 2B) as cells within the GCL containing hyperphosphorylated tau (Fig. 2C, D). An intensive AT8-positive staining was also detected in the axons of RGCs.

### Binding kinetics of FSB in vivo

**Figure 3:**
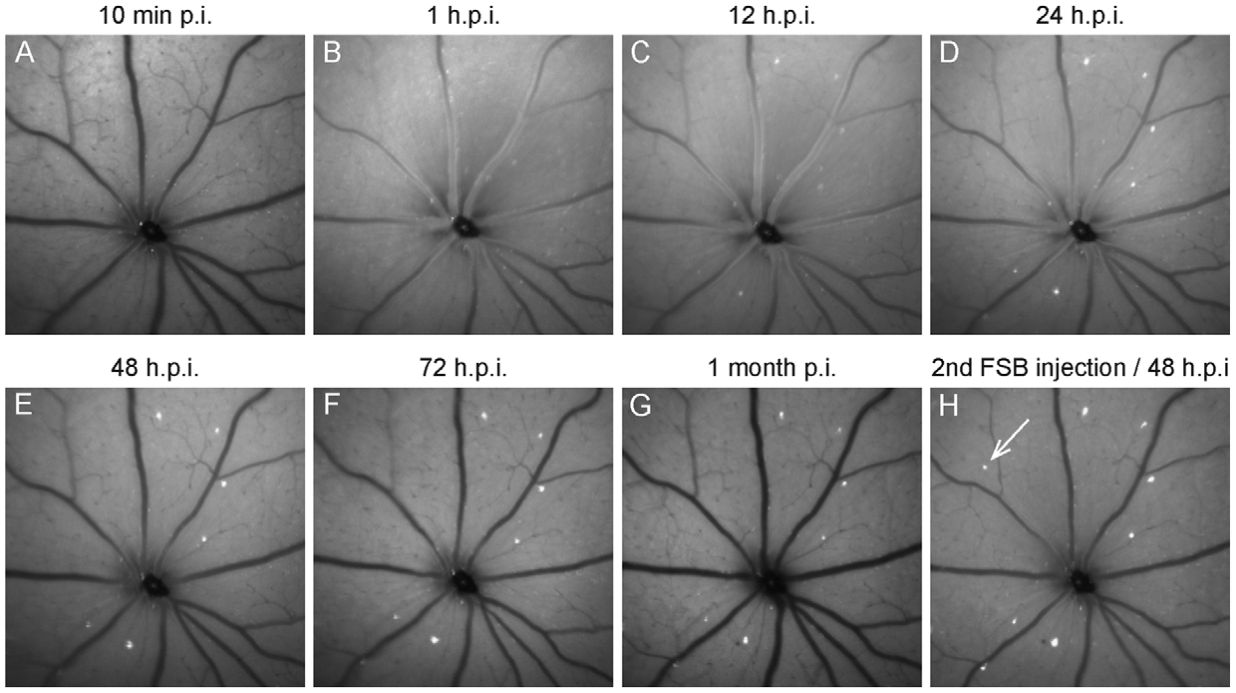
Binding kinetics of FSB. **A** 10 minutes after the systemic administration, no fluorescence of FSB was observed in SLO images obtained with 450 nm excitation. **B** Within retinal blood vessels, bright fluorescence appeared 60 min post-injection and was still present 12 hours post-injection (**C**). **D** 24 hours post-injection most of the fluorophore was cleared from the blood stream and distinct FSB-positive cells could be observed. **E–G** These FSB-positive cells were still present 48, 72 hours and even 1 month after the first injection of FSB. **H** New FSB-positive cells did not appear until a second FSB injection (arrow). p.i. post-injection.

To monitor the binding kinetics of FSB to fibrillar tau within the P301S retina in more detail *in vivo* images were obtained after a single FSB injection at 450 nm excitation. No signal could be observed in the retina 10 min after the injection (Fig. 3A), whereas bright fluorescence was prominent within the retinal blood vessels 1 hour and 12 hours post injection (h.p.i.) (Fig. 3B, C). 24 h.p.i. the fluorophore was almost completely cleared from the blood-stream and distinct FSB-positive cells could be distinguished from the background fluorescence (Fig. 3D). The observed FSB-positive cells were still fluorescent 48 h.p.i., 72 h.p.i. and even 1 month after the first injection without any sign of bleaching (Fig. 3E–G). New FSB-positive cells did not appear until a second injection of the fluorophore (Fig. 3H).

### In vivo long-term imaging of the formation of fibrillar tau aggregates

**Figure 4:**
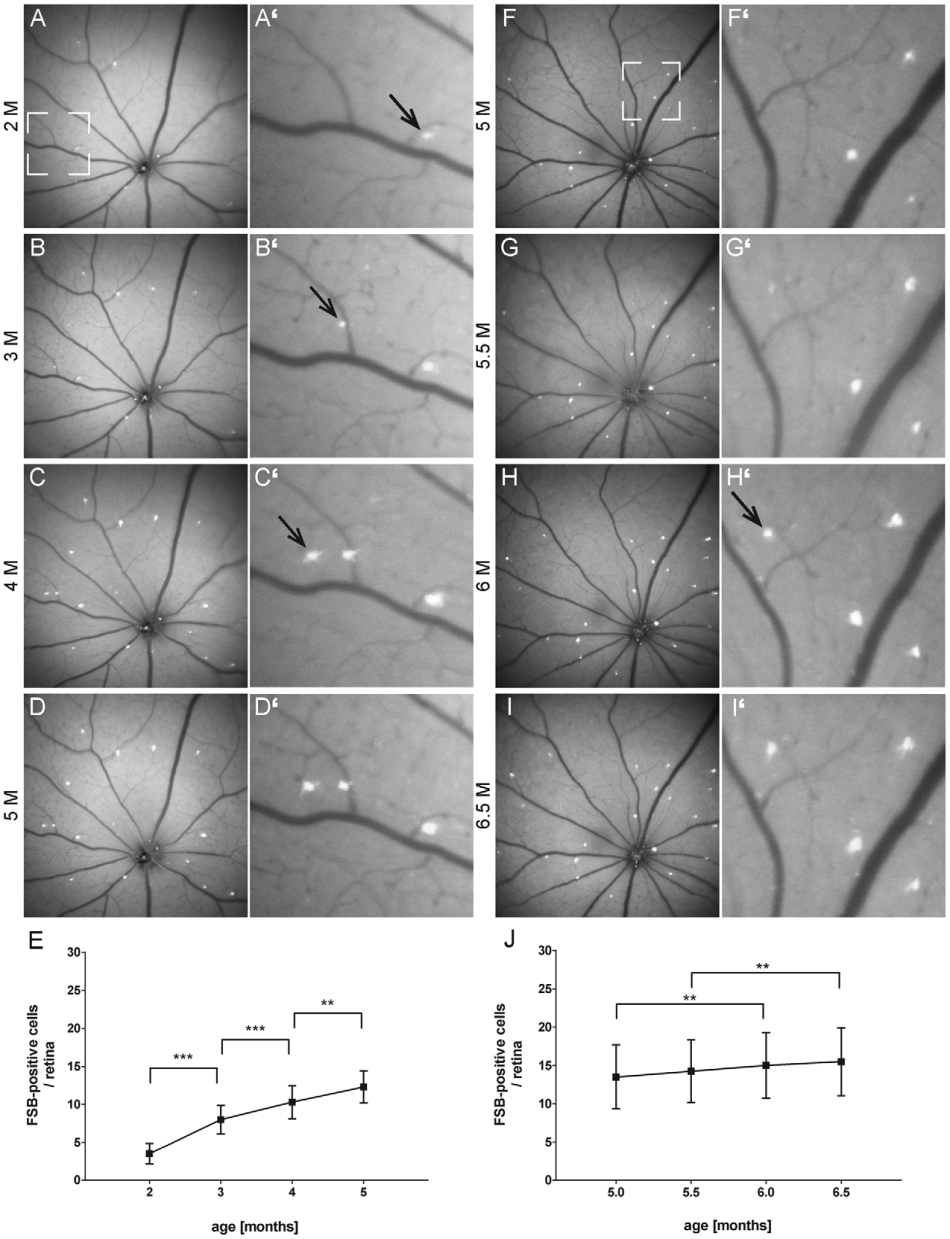
*In vivo* long term imaging of FSB-positive cells displays the disease progression in P301S mice. **A–D** Representative retina imaged between 2 and 5 months of age in an interval of 1 month. **F–I** Second retina imaged between 5 and 6.5 months of age in an interval of 2 weeks. **A'–I'** Enlarged images of the boxed areas in **A** and **F** demonstrating the appearance of new FSB-positive cells over the time (arrows). FSB was administrated systemically before each imaging session and images were acquired at 450 nm excitation. **E, J** Quantification of the increasing number of FSB-positive cells in the age from 2 to 5 months and 5 to 6.5 months. The curve displays the mean from 10 different retinas (n = 5 mice). Error bars show SD. **P<0.01; ***P<0.001.

The capability to detect fibrillar tau *in vivo* allowed us to follow up the progression of the tau pathology in the P301S retina over a long period of time. Homozygous P301S mice were scanned between 2 and 5 months of age in an interval of 1 month (n = 5) (Fig. 4 A–D). In a second experiment, P301S mice were imaged between 5 and 6.5 months of age in an interval of 2 weeks (n = 4) (Fig. 4 F–I). 24–48 hours before each image acquisition at 450 nm excitation, FSB was administered systemically. The precise repositioning of the animal in front of the optical lens was enabled by the custom-made mouse head holder (Fig. 1A). Thus, the same retinal region could be imaged repeatedly and new appearing FSB-positive cells were detected over the whole period of time (Fig. 4 A'–I'). For quantification, FSB-positive cells were discriminated from points of increased autofluorescence according to their spectral properties (Fig. 1 H, I). The quantification revealed a constant increase in the number of FSB-positive cells from 2 months (3.5±1.3, mean ± SD) to 5 months (12.3±2.1) of age (Fig. 4E). This steady growth in number of FSB-positive cells was still present in a second imaged cohort of aged mice from 5 (13.5±4.2) to 6.5 months (15.5±4.4) of age (Fig. 4J). Interestingly, no loss of cells after the formation of fibrillar tau was noticed. At the most advanced state of the tangle-formation in this mouse model (6.5 months) all FSB–positive cells could be counterstained in retinal whole mounts with the neuronal marker NeuN, confirming the viability of neurons containing fibrillar tau (Fig. S1 B–B'').

### Ex vivo analysis of the formation of fibrillar tau aggregates

**Figure 5:**
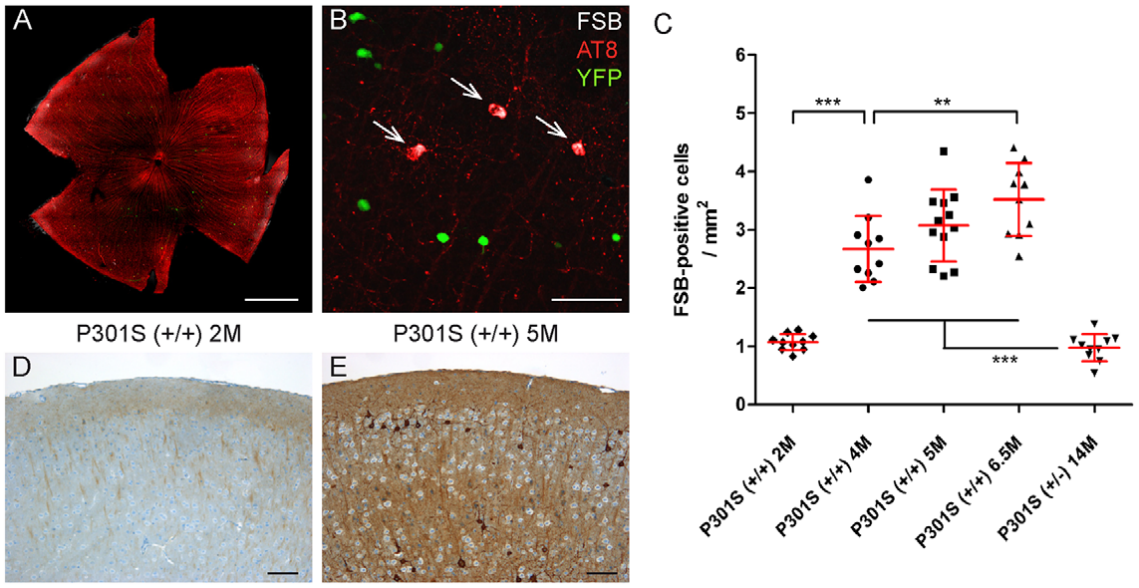
*Ex vivo* analysis of FSB-positive cells. **A, B** FSB-positive cells were counted in retinal whole mounts of P301S mice (red, AT8; white, FSB; green, YFP). **C** Quantification of FSB-positive cells of homozygous and heterozygous P301S mice in cohorts of different ages (n = 5–6). Each data point represents the number of FSB-positive cells in one retina normalized to the area. Shown are mean values ± SD. **P<0.01; ***P<0.001. **D, E** AT8-staining in the cortex of homozygous P301S mice at the age of 2 and 5 months. Scale bars: 1 mm (**A**), 100 µm (**B, D, E**).

FSB-positive cells were counted in retinal whole mounts of P301S-mice (Fig. 5A) in order to validate the *in vivo* detected constant increase of fibrillar tau aggregates. The *ex vivo* observed FSB-positive cells could all be counterstained with the antibodies AT8 (Fig. 5B) or AT100 (data not shown). Consistent with the *in vivo* data, a constant growth in the number of FSB-positive cells was found in homozygous animals (n = 5–6) between 2 months (1.1±0.1, mean ± SD), 4 months (2.7±0.6) and 5 months (3.1±0.6) of age (Fig. 5C). This increase in the number of FSB-positive cells was also detected in further aged animals (6.5 months; 3.5±0.6). In comparison, the retinas of heterozygous P301S mice at 14 months of age (n = 5) showed a far less developed tau pathology (1.0±0.2). Remarkably, the retinal pathology preceded the cortical pathology. Only weak immunoreactivity against hyperphosphorylated tau (antibody AT8) was detected in the cerebral cortex of 2-month-old animals (Fig. 5D), while numerous AT8-positive neurons and strong neuropil labelling could be found by the age of 5 months (Fig. 5E).

### Pathological alterations of tau and Aβ in the retinas of AD and PSP patients

**Figure 6:**
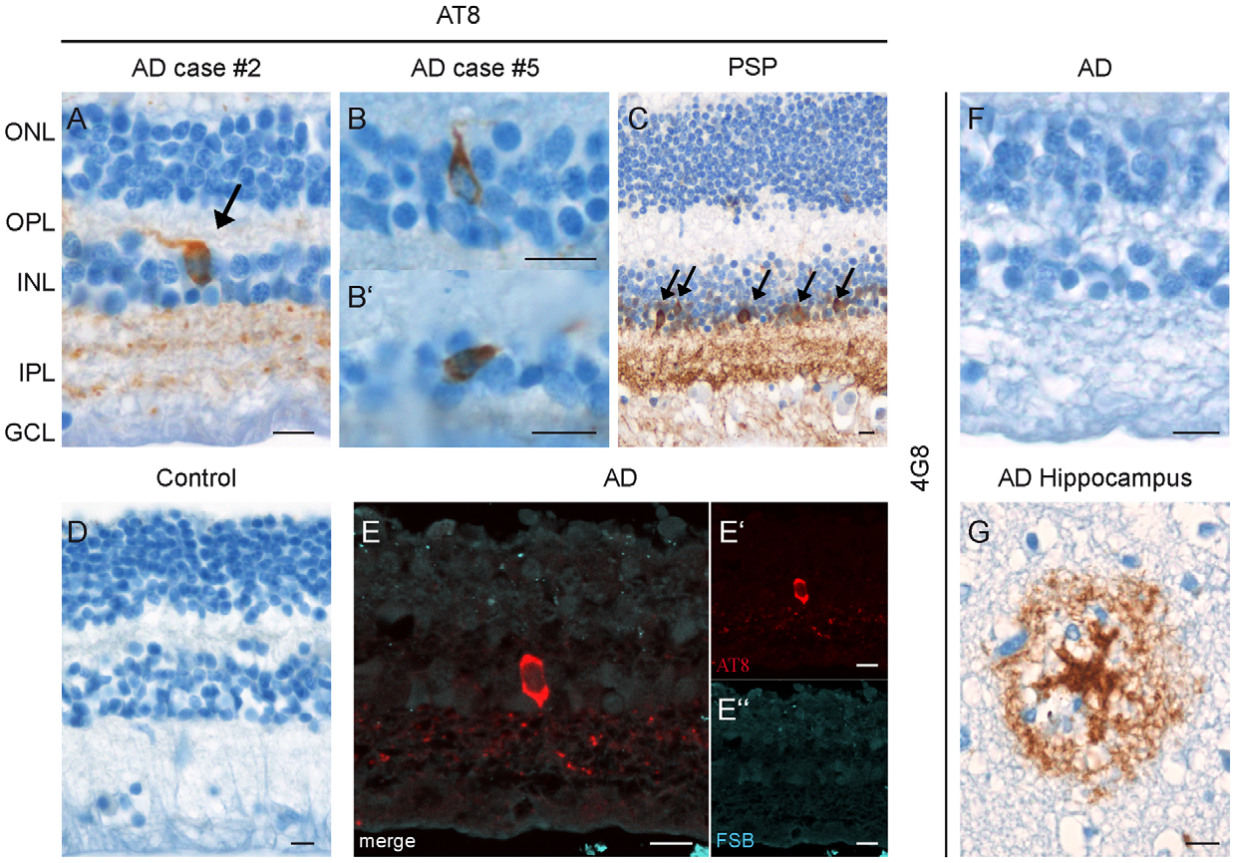
Pathological alterations of tau and Aβ in the retinas of human AD and PSP patients. **A–C** *Post mortem* retinas of AD and PSP patients showing AT8-positive cells in the INL (arrows) and stainings of the plexiform layers and GCL. **B, B'** Higher magnification image of individual AT8-positive cells in the INL. **D** Control retinas were not immunopositive for AT8. **E–E''** AT8-positive cells could not be co-stained with FSB in AD and PSP retinas. **F** No 4G8-positive staining against Aβ was found in the retinas of AD patients. **G** In comparison, control brain slices of AD patients showed severe 4G8-positive Aβ-plaques. ONL, outer nuclear layer; OPL, outer plexiform layer; INL, inner nuclear layer; IPL, inner plexiform layer; GCL, ganglion cell layer. AD, Alzheimer's disease; PSP, progressive supranuclear palsy. Scale bars: 10 µm.

To clarify whether the *in vivo* detection of fibrillar tau in the mouse retina can be translated to the diagnostic question concerning human tauopathies, we performed immunohistochemical stainings on paraffin-embedded retinas of patients, which had suffered from different tauopathies (Table 1). In this study, 5 out of 6 AD- and 2 examined PSP-cases showed AT8-positive inclusions of hyperphosphorylated tau in the retinas. Besides the plexiform layers and the GCL, cells in the inner nuclear layer (INL) were intensively stained by the antibody (Fig. 6 A–C). Remarkably, already very young patients with forms of familial Alzheimer's disease (Cases #1 and #5) revealed AT8-positive inclusions (Fig. 6 B, B'). In contrast, the retinas of control patients did not show any comparable staining (Fig. 6 D).

The observed inclusions of hyperphosphorylated tau could not be co-stained with the fluorophore FSB (Fig. 6 E–E''). Further attempts to confirm the presence of fibrillar tau aggregates using Thioflavin-S, Gallyas-Silver or the antibodies AT100, AT180, AT270 or PHF-1 did not result in any positive staining of the human retinas (data not shown). In addition, we performed histological stainings against Aβ, the second neuropathological hallmark of AD. No evidence was found for fibrillar accumulations of Aβ in the retinas from AD patients with the antibody 4G8 (Fig. 6 F) nor by using the fluorophores FSB and Thioflavin-S (data not shown). Brain slices of human AD patients were used to confirm the staining results.

## Discussion

In this study, we have monitored the development of retinal tau pathology on single cell level for the first time *in vivo*. Longitudinal laser scanning ophthalmoscopy in the P301S mouse model revealed an increase over several months in the number of RGCs that contain fibrillar, FSB-positive tau aggregates. Furthermore, we were able to demonstrate hyperphosphorylated but not fibrillar tau in the retinas of different human tauopathies.

Previous studies addressing the potential diagnostic value of AD-related pathology in the retina have mainly focused on Aβ-pathology in transgenic mouse models [14], [15], [16]. Koronyo-Hamaoui et al. first described retinal Aβ-plaques in human AD patients and showed that these aggregates can be imaged *in vivo* in the APP_swe_/PS1_ΔE9_ mouse model by the application of curcumin [13], [30]. In contrast, we could not detect any Aβ-plaques in the retinas of neuropathologically confirmed cases of AD. We cannot completely exclude that this discrepancy between our results and the study of Koronyo-Hamaoui et al. is based on technical differences in the immunohistochemical stainings. Whereas the previous study was performed on cryosections respectively retina whole mounts and used the primary 4G8 antibody in a 10 times higher concentration, we applied conventional immunohistochemical procedures on paraffin embedded material as used in the routine neuropathological diagnosis of AD. However, our findings are in accordance with previous studies that failed to detect Aβ-plaques within the retinas of neuropathologically confirmed AD cases [31], [32] and patients with a clinical presentation of AD [33]. Furthermore, we could not detect retinal Aβ-plaques *in vivo* in APP_swe_/PS1_ΔE9_ (n = 7, 16–24 months) and Tg2576 (n = 5, 16–22 months) mice by using the fluorophores FSB and BSc4090, both shown to bind to Aβ-plaques in the brain of these mice [34], [35]. As demonstrated in this paper as well as in a previous retina imaging study defining the nature of spots of increased autofluorescence in the mouse retina [27], any fluorescent signal in the retina has to be analysed in depth for specificity. Therefore, we always examined the retinas before the application of fluorophores like FSB or BSc4090, in order to be able to subtract unspecific signals. *Ex vivo* immunohistochemical studies using various Aβ-antibodies (4G8, 6E10, NAB228) on retinal serial sections of these mice and other AD mouse models (3xTg-AD [36], APPPS1 [37], TgCRND8 [38]) confirmed our *in vivo* observation that Aβ-plaques cannot be detected in the retina of these transgenic models even at very old age (unpublished observations). These negative findings are in accordance to similar attempts at the University of Pittsburgh (Dr. C.M. Mathis, personal communication).

In comparison, hyperphosphorylated tau could clearly be detected in the retinas of AD and PSP cases. Therefore, we focused our *in vivo* study on the detection of tau pathology in the mouse retina. We chose a transgenic mouse model expressing human tau with the P301S mutation, which develops abundant FSB-positive tau inclusions in the brain, spinal cord, and the retina [17], [18], [24].

We monitored the formation of fibrillar tau aggregates *in vivo* for the first time by non-invasive means. In future studies, this technique will be useful for the screening of drugs aimed at reducing the formation of fibrillar tau aggregates. Since individual animals can be studied longitudinally over several months, errors due to variations in the strength of the pathology between single mice can be reduced. Interestingly, RGCs harbouring FSB-positive inclusions survived over several months fitting to the observation that the overall ganglion cell number in the retina of P301S mice was not found to be reduced [18]. This indicates, similar to observations obtained in the cerebral cortex of other tau transgenic mouse lines [39], [40], that the formation of fibrillar tau alone may not be sufficient to cause neuronal death. On the other hand, tau hyperphosphorylation and aggregation result in reduced axonal transport in the optic nerve and an increased loss of RGCs after mild excitotoxic injury in P301S mice [20]. Observing the formation of fibrillar tau aggregates in the retina could be useful to further investigate the kinetic of RGCs loss after similar injuries *in vivo*. Furthermore, the herein described long-term binding stability of FSB could allow an additional, high-resolution analysis of fibrillar tau formation and its effect on cellular environment by time-stamp technology as already shown for cerebral Aβ-plaques in transgenic mice [41].

Despite the limited number of human cases in our study, we provide for the first time evidence of the presence of hyperphosphorylated tau in the retinas of AD and PSP patients. The used antibody AT8 is a well-accepted marker in routine diagnosis of AD and recognizes tau doubly phosphorylated at Ser202 and Thr205 [21]. Remarkably, other tau epitopes were not found to be phosphorylated as confirmed by the use of different antibodies (AT100: Ser212/Thr214; AT180: Thr231/Thr235; AT270: Thr181; PHF1: Ser396/404). This might explain why to date no other study has described hyperphosphorylated tau in the retina of different tauopathies. Only one out of 6 examined AD cases with the most prolonged *post mortem* interval (72 h) showed no AT8-positive staining. Therefore, we speculate that the *post mortem* interval may affect the stability of hyperphosphorylated tau, as has already been shown for other phosphoproteins [42]. It remains to be seen, if hyperphosphorylated tau occurs in the retina of other types of tauopathies (e.g. frontotemporal dementia) and whether this pathological alteration can be observed at a presymptomatic stage of the diseases. Moreover, retinal pathogenesis in different types of dementias could be influenced by the aggregation of other proteins like indicated by changes in the distribution pattern of synucleins in AD or dementia with Lewy bodies [43], [44]. Interestingly, accumulations of hyperphosphorylated tau were also found in the retina of glaucoma patients [45]. These findings tend to support a possible link between glaucoma and AD like discussed in the literature [46].

The presence of AT8-positive, hyperphosphorylated tau in the human retina led us to the hypothesis that our approach could be applicable in the diagnosis of human tauopathies. Histological examinations of the human retinas however revealed no evidence for fibrillar tau aggregates or Aβ-plaques. Molecular probes like FSB or Thioflavin-S bind to tau and Aβ in their fibrillar conformation only. Therefore, an ophthalmic detection of hyperphosphorylated tau based on the currently available fluorophores won't be applicable to human diagnosis. Our findings challenge the results of an earlier study that described the potential diagnostic value of Aβ-plaques in the retina of human AD cases [13], [30].

In conclusion, we developed a method which potentially could be used for the monitoring of compounds inhibiting the formation of fibrillar tau. We present hyperphosphorylated tau in the retinas of human tauopathies, but found no evidence for Aβ-plaques or fibrillar tau aggregates. According to these findings, Aβ and tau in the retina are of limited value for the proposed ophthalmic diagnosis of AD. A feasible diagnostic system based on the retinal tau-pathology would depend on the development of novel fluorescent probes against soluble, hyperphosphorylated tau.

## Supporting Information

Figure S1 **FSB-positive cells are vital.** **A–A''''** In P301S mice crossed with Thy1-YFPH, 1.9% of FSB-positive cells also contained YFP. No pathological alterations in the dendritic arbors of these cells could be observed. Shown is one co-labelled cell (FSB, AT8, and YFP) next to cells containing either YFP only or FSB and AT8 only. **A** x–y projection, **A''''** x–z projection. **B–B''** Cells containing fibrillar tau in the retina of aged P301S mice (6.5 months) are positive for NeuN (green, NeuN; white, FSB). Scale bars: 20 µm.
(TIF)
