# Supplementary figures and images for: Long-Term In Vivo Imaging of Fibrillar Tau in the Retina of P301S Transgenic Mice
Source: PLoS One. 2012 Dec 31;7(12):e53547. doi: 10.1371/journal.pone.0053547 (PMC3534024; doi:10.1371/journal.pone.0053547)

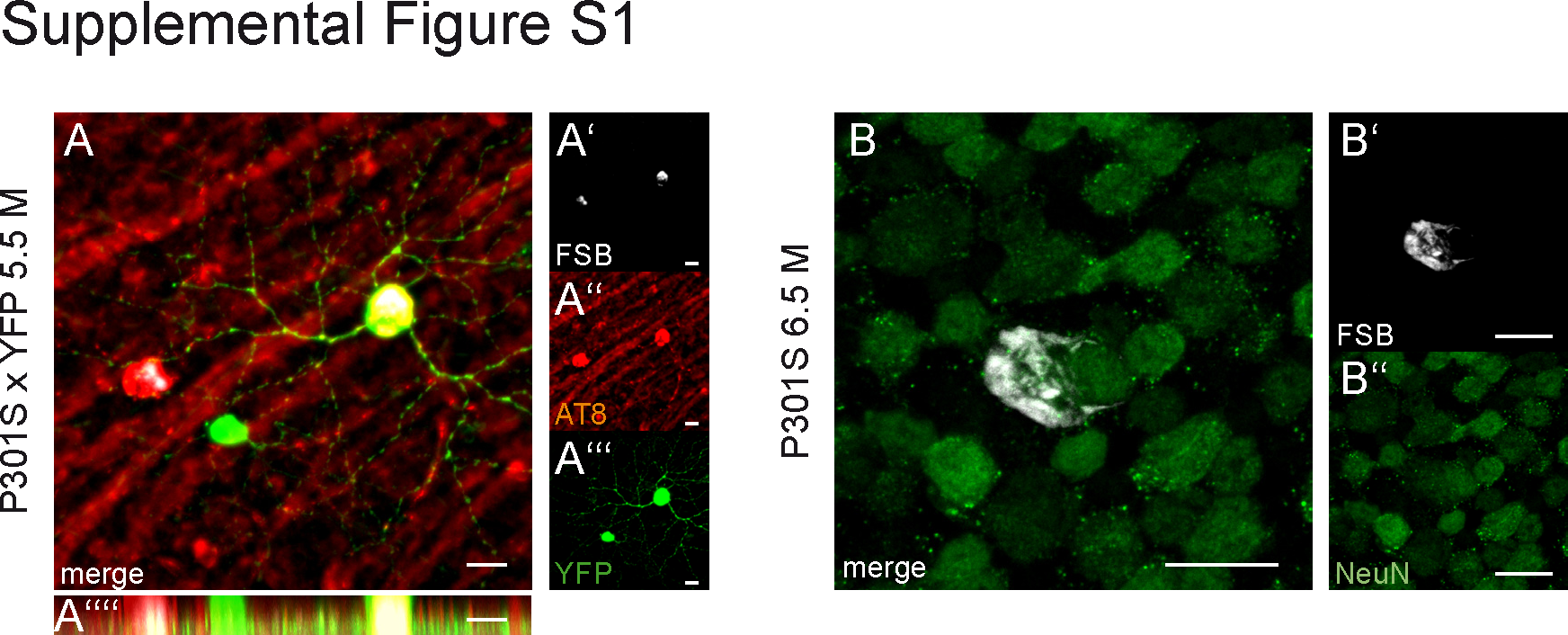

Supplement: Figure S1 — FSB-positive cells are vital. A–A'''' In P301S mice crossed with Thy1-YFPH, 1.9% of FSB-positive cells also contained YFP. No pathological alterations in the dendritic arbors of these cells could be observed. Shown is one co-labelled cell (FSB, AT8, and YFP) next to cells containing either YFP only or FSB and AT8 only. A x–y projection, A'''' x–z projection. B–B'' Cells containing fibrillar tau in the retina of aged P301S mice (6.5 months) are positive for NeuN (green, NeuN; white, FSB). Scale bars: 20 µm. (TIF) [file pone.0053547.s001.tif]
